# Effect of *Sempervivum tectorum* Extract on Some Biomarkers of Reproductive Function and Levels of Some Trace Elements in Male Rats Exposed to Aluminum

**DOI:** 10.3390/ani14081196

**Published:** 2024-04-16

**Authors:** Florin Muselin, Eugenia Dumitrescu, Alexandru O. Doma, Diana Maria Degi, Janos Degi, Jelena Savici, Catalin Cicerone Grigorescu, Diana Brezovan, Ioana Gencia, Romeo T. Cristina

**Affiliations:** 1 Faculty of Veterinary Medicine, University of Life Sciences “King Michael I”, 300645 Timisoara, Romania; florinmuselin@usvt.ro (F.M.);; 2 Working Group for Xenobiochemistry, Romanian Academy-Branch, 300645 Timisoara, Romania; 3 Faculty of Medicine Bucharest, Titu Maiorescu University, 300645 Timisoara, Romania; 4 Faculty of Medicine Timisoara, University of Medicine and Pharmacy ”Victor Babeș”, 300645 Timisoara, Romania

**Keywords:** *Sempervivum*, houseleek, aluminum, reproductive function, biomarkers, trace elements

## Abstract

**Simple Summary:**

Although aluminum was extensively studied in the last decades, questions still arise and present increasing interest regarding the reproductive toxicity, with the research still having lacunas, as was observed in a up-to-date and very ample review. In the present study, we show that aluminum is accumulating in the genital organs and also in sexual accessory glands, impairing the histoarchitecture of the reproductive system, except for bulbo-urethral glands and seminal vesicles, and it is negatively correlated with the hormone levels and the trace minerals from reproductive organs and accessory glands. At the same time, we observed that the use of *Sempervivum tectorum* aqueous extract can protect against deleterious effects of aluminum upon the main studied reproductive biomarkers. Some of the results are, for the first time, presented since we did not found research related to this, such as the aluminum content in the bulbo-urethral glands and seminal vesicles, the correlation between the trace minerals from the genital organs and sexual accessory glands and the hormone levels, and the correlation between aluminum level and the trace element levels in studied organs.

**Abstract:**

Aluminum, a contentious trace element found in the environment, has been demonstrated to have harmful effects on both humans and animals. In contrast, *Sempervivum tectorum*, an evergreen plant, has been found to offer numerous beneficial effects for both humans and animals. Therefore, this study aims to assess the protective effect of *S. tectorum* on certain reproductive biomarkers in male rats exposed to aluminum. Thirty-five Wistar rats were randomly divided into five groups: NTC (no-treatment control) received distilled water; NC (negative control) received drinking water containing 1 mg/L aluminum sulfate (AS); E1 received 1 mg/L AS along with an 8% *S. tectorum* extract; PC (positive control) received only 8% *S. tectorum* extract; E2 received 1 mg/L AS for three months followed by 8% extract for one month. The study analyzed testosterone, LH, FSH, body weight, and the histological structure of the testis, epididymis, and prostate, as well as the levels of zinc, manganese, copper, and iron in these organs. Significant decreases in body weight, testis, and epididymis size were observed in the aluminum-exposed groups compared to the control, whereas these decreases were not significant in the *S. tectorum*-treated groups compared to the control. Aluminum exposure led to significant decreases in testosterone and LH levels, with FSH levels showing a nonsignificant decrease in males, which were mitigated significantly by the administration of the plant extract. Histological analysis revealed alterations in the testis, epididymis, and prostate of the AS-exposed groups, including necrosis of seminiferous tubule epithelium and Leydig cells in the testis, and basal epithelial necrosis in the epididymis and prostate. Aluminum levels increased in all organs studied, while levels of zinc, copper, iron, and manganese decreased, showing a negative and significant correlation with aluminum levels. The aqueous extract of *S. tectorum* demonstrated a protective effect on certain studied biomarkers in male rats affected by aluminum exposure.

## 1. Introduction

Aluminum (Al) is a ubiquitous and abundant metal in the environment, placed as the third most common constituent of the Earth’s crust [1,2,3]. While initial studies on aluminum suggested potential biological significance, over the last two decades, numerous studies have contradicted this notion. Recent research has consistently demonstrated the lack of bio-utility of this metal, emphasizing its association with adverse effects on various target organs, including the kidneys [4], brain [5,6], and the male and female reproductive systems [3,7,8,9,10,11,12,13].

Excessive amounts of aluminum can result in its accumulation in specific organs, potentially causing damage to testicular tissues in both humans and animals. Elevated levels of aluminum in testes, Leydig cells, spermatozoa, seminal plasma, and blood have been linked to compromised sperm quality and viability [14]. 

The detrimental effects of aluminum are varied and can lead to a complex systemic toxicity. Its toxic actions target various molecular pathways within cells, disrupting cellular balance and resulting in damage that contributes to systemic toxicity, manifesting as structural and functional abnormalities in organs [15]. Primarily, aluminum’s toxic effects stem from its pro-oxidant properties, inducing oxidative stress, free radical damage, and the oxidation of cellular proteins and lipids [16].

Despite extensive investigations into aluminum, questions persist, particularly in the realm of reproductive toxicity. Existing research still exhibits gaps, as highlighted in a comprehensive and up-to-date review by Yokel [2]. 

*Sempervivum tectorum* L., an evergreen plant, known as the common houseleek, is a perennial herb with a dense basal rosette of fleshy sharp-tipped, obovate to oblong leaves having red-colored flowers arranged in terminal cymes [17,18,19]. The houseleek is a drought-resistant plant, native to central and southern Europe, growing on roofs, scree, and weathered rocks, traditionally used fresh rather than dried [18,20]. *S. tectorum* contains phytochemical compounds such as flavonoids, phenolic acids, and tannins, which possess antioxidant and metal-chelating properties [20,21,22]. These compounds have the potential to scavenge reactive oxygen species (ROS) generated by aluminum toxicity and inhibit the harmful effects of aluminum on the reproductive system. Aluminum-induced oxidative stress is considered a major cause of male reproductive disorders [7,8,9,10,11]. *S. tectorum* has been reported to exhibit significant antioxidant activity due to its phytochemical constituents [21]; the antioxidative capacity of *S. tectorum* can help mitigate the oxidative damage caused by aluminum exposure and protect against reproductive disorders [17]. 

Nowadays, the plant is linked to numerous effects, such as antinociceptive, liver protective, and membrane-stabilizing effects, which are believed to be due to the antioxidant activity of its highly phenolic content [21,23].

The aim of the present study was to underline the effect of the aqueous extract of *S. tectorum* on some morphological and biochemical markers of male reproductive function, as well as the correlation between aluminum level and the levels of sexual hormones and some trace minerals with importance for reproductive function.

## 2. Materials and Methods

### 2.1. Plant Extract

The *S. tectorum* freshly gathered leaves were separated from the rosette and roots, washed, and then dried in an oven at 50 °C until reaching a consistent weight. Extraction was carried out, in Erlenmeyer flasks, from the leaves with a particle size of 0.4 mm, using tap water (at 0.8/10 *w*/*v* ratio). The mixture was heated to 90 °C for 10 min in a water bath, filtered twice, and subsequently stored at 4 °C for a maximum duration of three days [17]. The chemical and mineral composition is presented as Appendix A.

### 2.2. Animals and the Experimental Protocol

Thirty-five six-month-old Wistar rats weighing 350 ± 15 g were utilized for the study, sourced from the authorized animal facility at the University of Medicine and Pharmacy “*Victor Babeş*” Timisoara. The rats were housed in five groups (n = 7) in standard polycarbonate cages (dimensions: length × width × height = 750 × 720 × 360 mm), maintaining a 12 h light/dark cycle and a controlled temperature of 23 ± 2 °C. They were fed a Biovetimix standard diet (Bioveti code 140-501, Romania, details in Appendix B) and acclimatized to the experimental conditions for one week before the start of the experiment. The handling of animals adhered to directive 2010/63/EU [24] and the guidelines of the National Research Council (NRC) [25].

The experimental period, lasting three months for all groups except E2, which spanned four months, received approval from the Ethical Committee of the University of Life Sciences “*King Michael I*” in Timisoara (No. 120/2018). Following the acclimatization period, the rats were randomly assigned to five groups:

- Group I (NTC—no-treatment control, received distilled water).
- Group II (NC—negative control) received 1 mg/L aluminum as aluminum sulfate (AS) in drinking water (1 mg/L being the level that demonstrated marked effects on reproductive function as were observed in a previous study) [3].
- Group III (E1 received 1 mg/L AS + 8% *S. tectorum* extract as drinking water).
- Group IV (PC—positive control received 8% *S. tectorum* as drinking water).
- Group V (E2 received 1 mg/L AS for three months, and thereafter, 8% *S. tectorum* for one month).

### 2.3. Samples Collection and Analysis

Daily food and water/*S. tectorum* intake in controls and experimental groups were monitored and are presented in Appendix C. 

At the end of the exposure period, the animals were euthanized by overdosing a combination of anesthetics, specifically 300 mg/kg.BW ketamine (ketamine 10%, CP Pharma, Burgdorf, Germany) and 30 mg/kg.BW xylazine (Narcoxyl, Intervet International, The Netherlands). Blood and organ samples were then collected for subsequent analyses. 

The blood was collected in a BD vacutainer (Serum Plain BD ref no. 367837), without anticoagulant, in order to obtain serum samples. The serum testosterone (T), luteinizing hormone (LH), and follicle stimulating hormone (FSH) levels were determined by chemiluminescence using the Randox Evidence Evolution Biochip Array (Randox Laboratories, UK); the analyses were performed by Tody Laboratories, Bucharest, Romania (Certified laboratory).

For histological examination, the genital organs and sexual accessory glands were washed in saline buffer, immersed and fixed in Bouin’s solution, and embedded in paraffin, then were sliced at 5 µm and stained by hematoxylin and eosin method. The histoarchitecture changes were studied using an Olympus CX 41 microscope (Olympus, Leicestershire, UK) with image capture software and data interpretation, at 200× magnification.

In order to analyze trace elements, the organs samples were prepared by microwave digestion (Multiwave GO, Anton Paar, GmbH, Graz, Austria) using nitric acid 10 mL and hydrogen peroxide 2 mL, which were added to a 0.5 g sample and submitted to the digester with the following parameters: 120 °C, 800 W for 20 min. The samples were analyzed by atomic absorption spectroscopy (AAS) using a Perkin Elmer Analyst 800 (Perkin Elmer Inc., Waltham, MA, USA), and the operating conditions were in accordance with the manufacturers’ recommendations for each element. The atomization was carried out in an air-acetylene flame (ratio 13.5:2) for Cu, Zn, Fe, and Mn, and graphite furnace for Al, using hollow cathode lamps as radiation source. The calibration standards were prepared from a Merck CertiPur ICP 1000 mg/L stock standard solution (Merk, Darmstadt, Germany).

### 2.4. Statistical Analysis

The results are presented as mean ± SEM (standard error of the mean) and were subjected to statistical analysis using one-way ANOVA with Bonferroni correction and Tukey’s post hoc test for group comparisons. Additionally, the Pearson correlation test was employed to assess the correlation between aluminum, hormone levels, and trace element concentrations. The statistical analyses were performed using Graph Pad Prism 9.1 for Windows (Graph Pad Software, version 9.1.2, San Diego, CA, USA), with significance set at *p* < 0.05 for all values.

## 3. Results

### 3.1. Body Weight and the Weight of Genital Organs and Sexual Accessory Glands

The total body weight (Table 1) was significantly (*p* < 0.01) decreased in rats exposed to aluminum compared to the control group (NC/NTC: +12.58%). The administration of *S. tectorum* extract led to a significant (*p* < 0.01) increase in body weight compared to the group that received only aluminum (E1/NC: +11.93%), reaching almost the weight of the control group (E2/C: −2.15%, *p* > 0.05). When the extract was administered for one month after aluminum exposure, the body weight was significantly higher (*p* < 0.05) comparative to rats exposed to aluminum (E2/E1: +9.05%), but still remained lower than in control (E2/C: −4.67%).

Testes (**TS**), epididymis (**EP**), bulbo-urethral glands (**BU**), and seminal vesicles (**SV**) weights were significantly lower in groups that received aluminum compared to control (NC/NTC: TS: −18.62%, EP: −53.16%, BU: −29.03%, SV: −16.03%), except for prostate (**PR**) weight, which was found to be significantly (*p* < 0.05) higher in the aluminum-exposed group compared to control (NC/NTC: +16.67%). In both sexual organs and sexual accessory glands (except prostate), in the group that received *S. tectorum* extract, the weight was higher than in the group exposed to aluminum, but not statistically significant (*p* > 0.05), even if an increase was observed (E1/NC: TS +9.95%, EP +21.62%; PR −23.46%; BU +27.27%; SV +10.01%), and although the weight was increased, it remained lower than in the control group (E1/NTC: TS −10.52%, EP −43.03%, *p* < 0.05; PR −10.71%; BU −9.67%; SV −7.63%). 

There were not significant (*p* > 0.05) differences in the group that received *S. tectorum* extract compared to control, but there were some significant differences when the extract was administered for one month after the aluminum administration compared to the group that received the extract and aluminum at the same time (E2/NC: TS +16.91%, EP +72,97%, PR −26.53%, BU +31.81%, SV +17.27%).

### 3.2. Biochemical Reproductive Biomarkers

**Figure 1:**
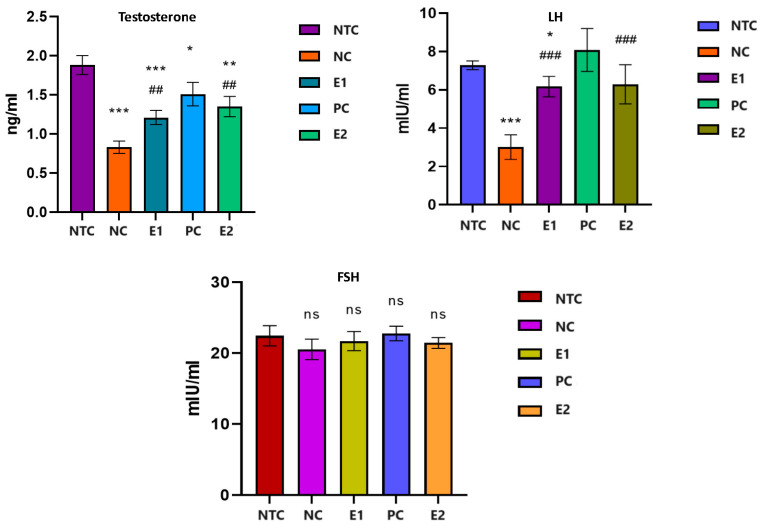
The levels of testosterone, LH, and FSH in rats exposed to aluminum and *S. tectorum* extract. Comparative to NTC: * *p* < 0.05, ** *p* < 0.01, *** *p* < 0.001; comparative to NC: ## *p* < 0.01, ### *p <* 0.001. NTC—no-treatment control, NC—negative control (1 mg/L Al), E1 (1 mg/L Al + 8% *S. tectorum* extract), PC—positive control (8% *S. tectorum* extract), E2 (1 mg/L Al for three months followed by 8% *S. tectorum* extract one month).

Following the statistical analysis of the results (Figure 1), we found that the serum testosterone level decreased strongly significantly (*p* < 0.001) in the rats from the groups that received aluminum compared to that of the individuals in the control group (NC/NTC: −56.11%). The administration of aqueous extract of *S. tectorum* led to a significant increase (*p* < 0.05) in the serum level of testosterone compared to individuals in the NC group (E1/NC: +45.78%). In individuals who received aqueous extract of *S. tectorum* one month after aluminum exposure, testosterone levels increased significantly (*p* < 0.01) compared to individuals in the aluminum-exposed group (E2/NC: +62.65%), but did not reach the level of the control group; they remained at a significantly (*p* < 0.05) lower level in comparison (E2/NTC: −28.19%).

Serum LH decreased significantly (*p* < 0.001) in individuals exposed to aluminum compared to those in the control group (NC/NTC: −58.76%). The application of the aqueous extract of *S. tectorum* had a notable impact (*p* < 0.05) on the LH level compared to the group exposed solely to aluminum, resulting in a significant increase (E1/NC: +104.98%). This increase brought the level close to that of the control group, albeit it was still significantly lower than the level recorded in the control group (E1/NTC: −15.24%). Administration for one month of *S. tectorum* extract after exposure to aluminum led to an increase in LH, which was significantly higher than in individuals exposed to aluminum alone (E2/NC: +108.97%), but did not reach the level of the control group (E2/NTC: −13.59%).

Regarding the level of serum FSH, we found not-significant fluctuations (*p* > 0.05) in all groups. 

### 3.3. Histological Structure

**Figure 2:**
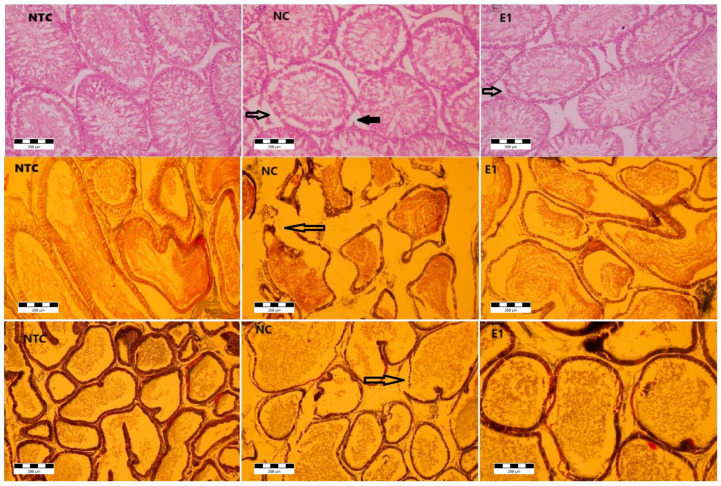
Histoarchitecture of testis, epididymis, and prostate in rats exposed to aluminum and *S. tectorum* extract (×200). Testis (first row NTC, NC, E1) 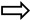
—seminiferous tubules epithelial necrosis; 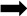
—Leydig cells necrosis; epididymis (second row NTC, NC, E1) 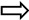
—epithelial basal necrosis; prostate (third row NTC, NC, E1); 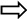
—epithelial basal necrosis. NTC—no-treatment control, NC—negative control (1 mg/L Al), E1 (1 mg/L Al + 8% *S. tectorum* extract).

Microscopic examination of testis (Figure 2) in the control group showed the apparently normal architecture, with the compact seminiferous tubules having a regular circumference. 

In the aluminum (NC) and aluminum plus *S. tectorum* extract (E1) groups, several microscopic changes in different degrees were noticed. The seminiferous tubules exhibited an altered appearance characterized by a thickened basement membrane, which, in certain instances, was disrupted. Additionally, there was a moderate to complete degeneration (necrosis) of the spermatogenic cells within some seminiferous tubules. If the seminiferous tubules are the main producers of sperm cells, the epididymis (Figure 2) is the androgen-dependent segment of male genital tract for sperm cells maturation and their storage until ejaculation. Under microscopic examination, the epididymis in the control group displayed a pseudo-stratified epithelium comprising diverse types of columnar cells. These cells were characterized by stereocilia that played a crucial role in maintaining the luminal microclimate essential for the maturation of sperm cells. 

Microscopic analysis revealed structural alterations in the epididymis of the NC and E1 groups, with varying degrees of severity. Disruptions in the epididymis duct wall were observed in specific areas, accompanied by the presence of atrophic cells within the epithelium. Furthermore, certain regions exhibited a hyperplastic epithelium. 

The prostate presents a tubulo-alveolar structure, with the alveoli being separated and supported by lamellae, made up of thin connective tissue. The epithelium is columnar with secretory cells and basal cells. For the control group, the microscopic examination revealed the normal structural appearance. For the NC and E1 groups, at the prostate level, morphological changes were noticed. Thus, the enlargement of spaces between alveoli suggests the interstitial edema. The alveoli were distorted with disruptions of the basement membrane. The epithelium was atrophied and hyperplasic in certain zones. 

There were no structural changes in the groups PC and E2, and there were also no changes observed in the bulbo-urethral glands and seminal vesicles in all groups. 

### 3.4. Trace Elements Content

The levels of aluminum (Al) showed a significant increase (Table 2) in all examined tissues of rats exposed to this element compared to the control (NC/NTC: **TS** +193.58%; **EP** +135.61%; **PR** +111.83%; **BU** +90.27%; **SV** +181.91%). In the group that received aluminum and plant extract, the levels decreased compared to those observed in the group exposed solely to aluminum. The decrease was statistically significant for the testes, epididymis, and prostate (E1/NC: **TS** −42.35%; **EP** −20.57%; **PR** −36.80%), and not statistically significant (*p* > 0.05) for the coagulating glands and seminal vesicles (**BU** −28.46%; **SV** −46.79%). Even though it decreased, the Al level remained higher than in the control group (E1/NTC: **TS** +69.23%; **EP** +87.12%; **PR** +33.87%; **BU** +36.11%; **SV** +50.00%).

Administration of plant extract for one month after the aluminum exposure proved to have a better effect regarding Al accumulation comparative to the group that received it together with the aluminum (E1/NC: **TS** −14.01%; **EP** −53.44%; **PR** −34.75%; **BU** +4.08%; **SV** −25.53%).

The examined trace elements demonstrated a similar pattern (Table 2), showing a decrease in both genital organs and sexual accessory glands of rats exposed to aluminum compared to the control.

The administration of *S. tectorum* extract exhibited a beneficial effect when administered concurrently with aluminum or for one month following aluminum exposure. This resulted in the restoration of trace element levels to a level nearly equivalent to those observed in the control group.

Upon correlating the levels of aluminum with the primary trace elements studied (Table 3), we observed a negative correlation between aluminum levels in genital organs and sexual accessory glands and those of zinc (Zn) and copper (Cu). This negative correlation was significant (*p* < 0.05) only in the testes, bulbo-urethral glands, and seminal vesicles for Zn, and in the testes for Cu. While iron (Fe) and manganese (Mn) levels were predominantly negatively correlated, there were some distinctions, with positive and nonsignificant (*p* > 0.05) correlations observed between Al levels in the epididymis for Fe and in the seminal vesicles for both Fe and Mn.

Also, we performed a statistical correlation between trace element levels and the reproductive biochemical biomarkers (Table 4), showing a significant (*p* < 0.05) negative correlation between aluminum and the levels of testosterone and LH, and a positive correlation between sexual hormones and Zn, Cu, Fe, and Mn, significantly (*p* < 0.05) mainly for the LH level and the levels of Cu and Fe. 

## 4. Discussion

The results of our study revealed that exposure to Al caused alterations in body weight, sexual organs, and sexual accessory glands biometry and histoarchitecture. Our study pointed out the significant decrease in testes and epididymis weight in rats exposed to 1 mg/L, compared to control. We think that the decrease in the body and sexual organs weights might be due to the mitochondrial dysfunction and a disruption in glucose metabolism [26] or it could be due to the decrease in testosterone level, which may result from the oxidative damage induced by Al, as suggested by Murshidi et al. [27] in male mice exposed to 1200 ppm aluminum chloride for twelve weeks that presented the same decrease in body weight, testes, and epididymis weight as this experiment. On the other hand, the decrease in the testosterone levels that were found in our study may explain the low epididymis weight that was observed in the Al-exposed animals; since the epididymis is an androgen-dependent organ, the weight could be linked to the action of testosterone on the epididymis epithelial cells [28].

Testes are specialized organs whose basic function is to produce germ cells and steroid hormones. LH stimulates Leydig cells in males to synthesize and secrete testosterone; these levels are regulated and controlled by the negative testosterone feedback, and accordingly, decreased testosterone will cause LH levels to increase through pituitary stimulation [29,30]. In our study we observed significant decrease in the testosterone level and LH level, but no influence on the FSH level, denoting a lack of pituitary response. In accordance with our findings, Sun et al. [30] noted the decrease in testosterone and LH without affecting FSH level in middle-dose and high-dose aluminum-exposed rats, concluding that Al exposure suppressed T and LH secretion and decreased androgen receptor protein and mRNA expression, which weaken the binding of androgen with the androgen receptor. The stability of FSH levels may be attributed to the fact that any alterations observed were within a compensatory range [30].

In a prior investigation, we observed a strong antioxidant effect in the *S. tectorum* aqueous extract when addressing Al-induced oxidative stress [17]. 

Guo et al. [31] documented that aluminum exposure in mice, specifically through aluminum chloride at 34 mg/kg.BW/intraperitoneal, heightened the activity of nitric oxide synthase, leading to an increased nitric oxide presence in the testis. Nitric oxide is recognized for its potential to regulate androgen synthesis, as highlighted in the study [32]. 

This association suggests a plausible explanation for the decline in testosterone levels, given that oxidative stress is acknowledged to disrupt endocrine processes and impede testosterone production [33]. Several studies have consistently indicated that aluminum (Al) can lead to damage in the testes and epididymis, resulting in hormonal imbalances and fertility issues [3,34,35], thereby supporting our findings. 

For example, Yousef et al. [36] conducted a study on rats exposed to 70 mg/kg.BW aluminum oxide nanoparticles, revealing detrimental effects on reproductive function. They observed decreases in body, testes, and epididymis weight, an increase in prostate weight, reduced testosterone levels, and alterations in testicular tissue structure, aligning with our findings observed at levels of 1 mg/L. However, their study reported increased levels of luteinizing hormone (LH) and follicle-stimulating hormone (FSH), which contrasted with our results.

Conversely, Moselhy et al. [37], in rats administered 34 mg/kg.BW aluminum chloride daily for sixty days, and Shahraki et al. [38], in rats injected with aluminum chloride into the lateral ventricle for twenty days, noted significant decreases in LH and FSH levels, partially corroborating our findings, where the FSH level remained within the compensatory range in our experiment. In contrast to our findings and those of the aforementioned authors, Mayyas et al. [39] reported an increase in testosterone and LH in male mice exposed to 1000 to 1400 ppm/day aluminum chloride in drinking water for 12 weeks. 

The present study’s histological assessments consistently revealed adverse impacts on the reproductive system in the testis, epididymis, and prostate. For instance, da Silva Lima et al. [12] documented disruptions in part of the seminiferous tubules within the testes, epithelial hyperplasia, and the accumulation of lipofuscin granules in secretory luminal cells within the prostate of gerbils treated with 10 mg/kg.BW AlCl3 for 30 days. Interestingly, these specific effects were not observed in our study with rats exposed to 1 mg/L Al. Furthermore, rats administered *S. tectorum* extract exhibited an improvement in the histological impairments observed in Al-exposed rats.

However, Martinez et al. [40] indicated that while Al impaired testis histology, it did not affect epididymis structure. Their study also noted alterations in body mass, genital organ weight, and sexual accessory gland weight in rats receiving AlCl3 at 100 mg/kg.BW for 60 days.

In the present study, we observed a restoration in the histological structure of organs, hormonal levels, and other parameters studied when utilizing *S. tectorum* aqueous extract. While we could not find direct research on the use of houseleek *S. tectorum* to counteract aluminum’s reproductive effects for comparison, there are studies that obtained results akin to ours by employing other extracts or substances possessing antioxidant properties. For instance, Mohammad et al. [41] observed that AlCl_3_ significantly decreased serum testosterone, LH, FSH, testicular weight, zinc levels, and Leydig cell count, all of which were restored through the use of coenzyme Q10 and fish oil, aligning with our findings. 

Olarewaju et al. [42] reported impaired reproductive function, noting a decrease in luteinizing hormone and an increase in FSH, leading to a substantial reduction in testosterone levels. They also observed degenerative changes in the testicular structure and Leydig cells in rats exposed to 300 mg/kg.BW aluminum chloride. These changes were mitigated by administering 200 mg/kg.BW quercetin over a 21-day exposure period. Similarly, Odo et al. [43] achieved positive outcomes in testosterone levels and the restoration of testicular structure using an ethanolic extract of *Citrullus lanatus* in rats treated with 100 to 200 mg/kg.BW aluminum chloride. This illustrates how various plants can protect against aluminum-induced reproductive harm, akin to what we observed in our study using *S. tectorum* extract.

A substantial body of research [3,7,8,9,11,17,27,32,36,38,39,40,41,42,43] has highlighted a significant increase in aluminum (Al) levels in the testes, epididymis, prostate, seminal vesicles, and bulbo-urethral glands compared to control groups, supporting our findings. However, it is important to note that we did not find studies assessing Al levels in sexual accessory glands for comparison.

Upon analyzing the correlation matrix, we noted a negative correlation between the Al level and sexual hormones, as well as important trace elements crucial for reproductive function, such as Zn, Cu, Fe, and Mn, in nearly all organs studied, except for Fe in the epididymis and Fe/Mn in seminal vesicles. Additionally, we identified a positive correlation between all examined trace elements and sexual hormone levels. These trace elements play essential roles as cofactors in enzymes involved in responding to oxidative stress, participating in various enzymatic reactions to counteract harmful reactive oxygen species [31,36].

In our current study, we observed a decrease in Zn levels among the Al-exposed groups, with a significant increase noted upon the administration of *S. tectorum*. Numerous studies have highlighted the vital role of Zn in regulating oxidative stress within the reproductive system, closely tied to various dehydrogenase activities [44]. Zn serves as a defender against free radicals and lipid peroxidation, thereby sustaining the proper functioning of antioxidant enzymes [45]. As previously mentioned, oxidative stress has been implicated in many reproductive system structural and functional impairments, a factor we believe was at play in our study.

Iron also holds significance in oxidative stress dynamics. Elevated concentrations in testicular tissue have been linked to antioxidant depletion, potentially escalating oxidative damage in rat testes [46], potentially elucidating part of our findings.

On another note, Zhu et al. [33], in a sub-chronic study on Al-exposed rats, proposed that reduced spermatogenesis and male reproductive disorders were primarily due to decreased testicular enzyme activity and imbalanced concentrations of other trace elements (Zn, Fe, Cu). They observed increased Al and Cu levels alongside decreased Fe and Zn content, suggesting that aluminum disrupted testicular energy and upset the trace element equilibrium within the testes, aligning with our study’s observations.

## 5. Conclusions

Drawing from our observations, which align with those of other researchers in this field, we can conclude that aluminum accumulation occurs in the genital organs and sexual accessory glands. This accumulation detrimentally affects the histological structure of the reproductive system, with exceptions noted in the bulbo-urethral glands and seminal vesicles. Furthermore, aluminum shows a negative correlation with hormone levels and the trace minerals present in reproductive organs and accessory glands. Concurrently, our findings suggest that the administration of *S. tectorum* aqueous extract can offer protection against the harmful effects of aluminum on the key reproductive biomarkers studied. Complementary investigations are required, incorporating additional measurements conducted throughout the experimental phase, to track the progression and identify the point at which the efficacy of the *S. tectorum* extract initiates.

## Figures and Tables

**Table 1:** The total body weight and the weight of genital organs and sexual accessory glands in rats exposed to aluminum and *S. tectorum* extract.

Specification	Groups				
	NTC (n = 7)	NC (n = 7)	E1 (n = 7)	PC (n = 7)	E2 (n = 7)
Body weight (g)	278 ± 2.15	243 ± 2.22 **	272 ± 3.18 ^##^	281 ± 2.39	265 ± 3.15 *^,#^
Testis (g)	2.47 ± 0.08	2.01 ± 0.09 *	2.21 ± 0.07	2.59 ± 0.16	2.35 ± 0.18 ^#^
Epididymis (g)	0.79 ± 0.02	0.37 ± 0.03 ***	0.45 ± 0.04 *	0.81 ± 0.04	0.64 ± 0.03 ^##^
Prostate (g)	0.84 ± 0.03	0.98 ± 0.02 *	0.75 ± 0.03 ^#^	0.86 ± 0.03	0.72 ± 0.02 **^,##^
Bulbo-urethral glands (g)	0.31 ± 0.01	0.22 ± 0.04 *	0.28 ± 0.08	0.36 ± 0.04	0.29 ± 0.03 ^#^
Seminal vesicles (g)	1.31 ± 0.02	1.10 ± 0.03 *	1.21 ± 0.11	1.42 ± 0.13	1.29 ± 0.14

Comparative to NTC: * *p* < 0.05, ** *p* < 0.01, *** *p* < 0.001; comparative to NC: ^#^ *p* < 0.05, ^##^ *p* < 0.01. NTC—no-treatment control, NC—negative control (1 mg/L Al), E1 (1 mg/L Al + 8% *S. tectorum* extract), PC—positive control (8% *S. tectorum* extract), E2 (1 mg/L Al for three months followed by 8% *S. tectorum* extract one month).

**Table 2:** Trace element levels in genital organs and sexual accessory glands of rats exposed to aluminum and *S. tectorum* extract (mean ± SEM).

Organs	Groups	Studied Trace Elements (µg/g)				
		Al	Zn	Cu	Fe	Mn
*Testis*	NTC (n = 7)	3.12 ± 0.07	143.21 ± 9.41	7.56 ± 0.93	164.29 ± 16.39	1.12 ± 0.15
	NC (n = 7)	9.16 ± 0.94 ***	98.18 ± 7.32 **	4.25 ± 0.74 **	111.42 ± 12.12 ***	0.82 ± 0.04
	E1 (n = 7)	5.28 ± 0.69 **^,##^	112.24 ± 5.28 *	6.35 ± 1.12 ^#^	134.89 ± 16.87 *^,#^	1.08 ± 0.07
	PC (n = 7)	2.17 ± 0.18 *	139.47 ± 6.38	8.22 ± 0.98	183.28 ± 21.22	1.31 ± 0.09
	E2 (n = 7)	4.54 ± 0.57 *^,###,$^	141.58 ± 11.25 ^#^	6.89 ± 0.65 ^#^	145.87 ± 12.28 *^,#^	1.29 ± 0.12 ^#^
*Epididymis*	NTC (n = 7)	1.32 ± 0.04	42.56 ± 3.87	2.84 ± 0.15	107.65 ± 12.15	0.58 ± 0.05
	NC (n = 7)	3.11 ± 0.45 **	28.42 ± 3.63 **	1.65 ± 0.08 *	109.58 ± 16.18	0.49 ± 0.11
	E1 (n = 7)	2.47 ± 0.61 *^,#^	39.51 ± 2.76 ^#^	1.91 ± 0.11 *	108.24 ± 18.27	0.51 ± 0.15
	PC (n = 7)	0.98 ± 0.06	40.22 ± 5.13	2.25 ± 0.09	106.29 ± 15.55	0.55 ± 0.09
	E2 (n = 7)	1.15 ± 0.09 ^##,$^	38.65 ± 5.29 ^#^	1.98 ± 0.08 *	108.97 ± 14.86	0.52 ± 0.08
*Prostate*	NTC (n = 7)	2.45 ± 0.14	136.22 ± 8.92	0.94 ± 0.08	75.88 ± 11.35	0.91 ± 0.16
	NC (n = 7)	5.19 ± 0.68 **	128.48 ± 7.55 *	0.88 ± 0.04	73.27 ± 16.28	0.88 ± 0.09
	E1 (n = 7)	3.28 ± 0.35 *^,##^	131.54 ± 6.88	0.91 ± 0.03	73.98 ± 12.54	0.87 ± 0.06
	PC (n = 7)	1.99 ± 0.64 *	132.81 ± 9.11	0.91 ± 0.04	74.29 ± 14.96	0.85 ± 0.19
	E2 (n = 7)	2.14 ± 0.58 ^##,$$^	130.11 ± 8.26	0.89 ± 0.05	72.51 ± 12.57	0.93 ± 0.16
*Bulbo-urethral glands*	NTC (n = 7)	0.72 ± 0.08	24.3 ± 6.25	1.12 ± 0.11	68.59 ± 8.45	0.37 ± 0.08
	NC (n = 7)	1.37 ± 0.19 *	18.5 ± 3.38 *	0.84 ± 0.02 *	66.74 ± 5.89	0.33 ± 0.07
	E1 (n = 7)	0.98 ± 0.07	21.2 ± 4.48	0.96 ± 0.08	65.38 ± 6.22	0.34 ± 0.06
	PC (n = 7)	0.52 ± 0.07	25.31 ± 6.55	0.93 ± 0.09	67.29 ± 7.88	0.34 ± 0.09
	E2 (n = 7)	1.02 ± 0.09 *	20.35 ± 8.11 *^,#^	0.93 ± 0.05	66.81 ± 6.34	0.36 ± 0.04
*Seminal vesicles*	NTC (n = 7)	0.94 ± 0.08	20.18 ± 3.45	4.81 ± 0.18	115.38 ± 12.86	0.64 ± 0.03
	NC (n = 7)	2.65 ± 0.64 ***	16.47 ± 1.12 **	4.22 ± 0.11	121.86 ± 19.97	0.63 ± 0.02
	E1 (n = 7)	1.41 ± 0.14 *	18.39 ± 2.15 *	4.39 ± 0.15	119.41 ± 12.55	0.65 ± 0.08
	PC (n = 7)	0.59 ± 0.09	19.58 ± 3.28	4.28 ± 0.18	118.28 ± 13.65	0.62 ± 0.04
	E2 (n = 7)	1.05 ± 0.18 ^#^	18.66 ± 4.45 ^#^	4.35 ± 0.15	120.55 ± 16.21	0.60 ± 0.08

Comparative to NTC: * *p* < 0.05, ** *p* < 0.01, *** *p* < 0.001; comparative to NC: ^#^ *p* < 0.05, ^##^ *p* < 0.01, ^###^ *p <* 0.001; comparative to E1: ^$^ *p* < 0.05, ^$$^ *p* < 0.01. NTC—no-treatment control, NC—negative control (1 mg/L Al), E1 (1 mg/L Al + 8% *S. tectorum* extract). PC—positive control (8% *S. tectorum* extract), E2 (1 mg/L Al for three months followed by 8% *S. tectorum* extract one month).

**Table 3:** Pearson *r* and *p* values of aluminum levels in genital organs and sexual accessory glands in correlation matrix with trace element levels.

Al Level in	Specification	Studied Trace Elements			
		Zn	Cu	Fe	Mn
*Testis*	*r*	−0890	−0.999	−0.963	−0.878
	*p*	0.043	0.0003	0.008	0.050
*Epididymis*	*r*	−0.780	−0.661	0.668	−0.755
	*p*	0.120	0.224	0.218	0.140
*Prostate*	*r*	−0.604	−0.534	−0.248	−0.193
	*p*	0.281	0.353	0.687	0.756
*Bulbo-urethral gl.*	*r*	−0.981	−0.584	−0.448	−0.405
	*p*	0.003	0.301	0.450	0.499
*Seminal vesicles*	*r*	−0.930	−0.399	0.678	0.220
	*p*	0.022	0.505	0.208	0.722

**Table 4:** Correlation matrix between hormones and testis trace element levels of rats exposed to aluminum and *S. tectorum* extract.

Hormones	Specification	Studied Trace Elements				
		Al	Zn	Cu	Fe	Mn
*Testosterone*	*r*	−0.877	0.871	0.863	0.799	0.648
	*p*	0.050	0.054	0.059	0.104	0.236
*LH*	*r*	−0.997	0.859	0.995	0.958	0.859
	*p*	0.001	0.061	0.004	0.010	0.062
*FSH*	*r*	−0.864	0.684	0.845	0.819	0.530
	*p*	0.058	0.202	0.071	0.089	0.358

## Data Availability

Data are available at: https://www.preprints.org/manuscript/202404.0443.

## Appendix A

**Table A1:** Chemical and mineral content of *S. tectorum* extract.

Composition	Unit	Content Value
Polyphenols	%	0.621
Tannins	%	0.169
Proanthocyanidins	%	0.122
Flavonoids (O-glycosides)	%	0.137
Flavonoids (C-glycosides)	%	0.195
Anthocyanidins	%	0.019
Al	mg/kg	92.14
Ca	mg/kg	82,351.01
P	mg/kg	2431.66
Mn	mg/kg	316.15
Zn	mg/kg	195.28
Fe	mg/kg	36.45
Mg	mg/kg	7258.14
Cu	mg/kg	1.85
Ni	mg/kg	0.59
K	mg/kg	32,574.11

## Appendix B

**Table A2:** Composition of standard rat diet according to producer.

Composition	Unit	Content Value
Protein	%	15
Fat	%	10
Cellulose	%	8
Ash	%	3
Ca	%	0.5
P	%	0.4
Vit. A	IU/kg	8500
Vit. D3	IU/kg	1500
Vit. E	mg/kg	20
Mn	mg/kg	52
Zn	mg/kg	30
Fe	mg/kg	40
Mg	mg/kg	55
Cu	mg/kg	8
I	mg/kg	1.5
Se	mg/kg	0.1

## Appendix C

**Table A3:** Daily food and water/*S. tectorum* intake in controls and experimental groups.

Specification	Groups				
	NTC (n = 7)	NC (n = 7)	E1 (n = 7)	PC (n = 7)	E2 (n = 7)
Food intake (g)	25.8 ± 1.15	25.3 ± 1.54	25.1 ± 1.18	24.9 ± 1.22	24.5 ± 1.65
Water/*S. tectorum* extract intake (mL)	29.2 ± 0.12	30.1 ± 1.03	29.5 ± 0.52	29.1 ± 0.6	28.8 ± 0.55

NTC—no-treatment control, NC—negative control (1 mg/L Al), E1 (1 mg/L Al + 8% *S. tectorum* extract), PC—positive control (8% *S. tectorum* extract), E2 (1 mg/L Al for three months followed by 8% *S. tectorum* extract one month).
